# Home advantage in the Winter Paralympic Games 1976–2014

**DOI:** 10.1007/s11332-017-0365-6

**Published:** 2017-06-06

**Authors:** Darryl Wilson, Girish Ramchandani

**Affiliations:** 1 Sheffield Hallam University, Academy of Sport and Physical Activity, A129 Collegiate Hall, Collegiate Crescent, Sheffield, S10 2BP UK; 2 Sheffield Hallam University, Sport Industry Research Centre, A118 Collegiate Hall, Collegiate Crescent, Sheffield, S10 2BP UK

**Keywords:** Home advantage, Disability sport, Performance, Paralympics, Olympics

## Abstract

**Purpose:**

There is a limited amount of home advantage research concerned with winter sports. There is also a distinct lack of studies that investigate home advantage in the context of para sport events. This paper addresses this gap in the knowledge by examining home advantage in the Winter Paralympic Games.

**Methods:**

Using a standardised measure of success, we compared the performances of host nations at home with their own performances away from home between 1976 and 2014. Both country level and individual sport level analysis is conducted for this time period. Comparisons are also drawn with the Winter Olympic Games since 1992, the point from which both the Winter Olympic Games and the Winter Paralympic Games have been hosted by the same nations and in the same years.

**Results:**

Clear evidence of a home advantage effect in the Winter Paralympic Games was found at country level. When examining individual sports, only alpine skiing and cross country skiing returned a significant home advantage effect. When comparing home advantage in the Winter Paralympic Games with the Winter Olympic Games for the last seven host nations (1992–2014), we found that home advantage was generally more pronounced (although not a statistically significant difference) in the case of the former.

**Conclusion:**

The causes of home advantage in the Winter Paralympic Games are unclear and should be investigated further.

## Introduction

There is a generally well-established body of academic literature that investigates the phenomenon of home advantage in sport. Courneya and Carron reviewed studies that documented the extent of the home advantage and concluded that it exists in major team sports [1]. They went on to develop a conceptual framework for home advantage research, according to which ‘performance’ is a function of: game location (i.e. home or away); game location factors that differentially impact on teams competing at home or away from home; and the critical psychological and behavioural states of competitors, coaches and officials. A subsequent review by Carron, Loughead and Bray proposed a slightly revised conceptual framework [2]. Table 1 compares the components of the two models. There are two major differences between the original model and the revised model. First, ‘officials’ were excluded from the latter, not because they do not potentially contribute to home advantage but as, unlike competitors and coaches, they do not have a designated home or visitor status. Second, the revised model incorporated the critical physiological factors of competitors and coaches (e.g. testosterone and jet lag).

**Table 1:** Conceptual framework for home advantage research

Component	Original model	Revised model
Game location		
Home	✓	✓
Away	✓	✓
Game location factors		
Crowd	✓	✓
Learning/familiarity	✓	✓
Travel	✓	✓
Rules	✓	✓
Critical psychological states		
Competitors	✓	✓
Coaches	✓	✓
Officials	✓	✖
Critical physiological states		
Competitors	✖	✓
Coaches	✖	✓
Critical behavioural states		
Competitors	✓	✓
Coaches	✓	✓
Officials	✓	✖
Performance outcomes		
Primary	✓	✓
Secondary	✓	✓
Tertiary	✓	✓

More recently Jamieson conducted a meta-analysis of studies on home advantage [3] and suggested that theoretical models would benefit from the inclusion of game-context factors, specifically when a contest occurs (time era effects) and what attributes are associated with particular contests (season length effects and sport effects), which may directly feed into game location factors. Differences in the magnitude of the home advantage between sports, and within sports over time, were also identified previously by Pollard and Pollard [4] when considering professional team sports in North America (American football, baseball, basketball, ice hockey) and England (football). With respect to individual sports, a review by Jones found mixed evidence for home advantage in comparison with the more robust evidence of its presence in team sports [5].

A subset of home advantage research is concerned with international multi-sport events, although these studies are rarely cited, or analysed as a separate category, in major literature reviews. For the most part, the Summer Olympic Games have been at the heart of previous research efforts [6–12]. A limited number of studies to date have examined home advantage in the context of the Winter Olympic Games [12–14] and the Commonwealth Games [15–17]. However, within home advantage research in general and its investigation within multi-sport events more specifically, there is a distinct lack of studies in relation to sports events that are targeted at elite athletes with a disability such as the Paralympic Games. It is this gap in the scientific knowledge that this paper attempts to address by focussing on the Winter Paralympic Games. To date, there have been 11 editions of the Winter Paralympic Games from 1976 to 2014. Nine different nations have hosted the competition in this time frame: Sweden (1976); Norway (1980 and 1994); Austria (1984 and 1988); France (1992); Japan (1998); USA (2002); Italy (2006); Canada (2010); Russia (2014). Between 1976 and 2014, the programme of the Winter Paralympic Games has incorporated six different sports: para alpine skiing (1976–2014); para cross country skiing (1976–2014); para biathlon (1988–2014); ice sledge speed skating (1980–1988 and 1994–1998); ice sledge hockey (1994–2014); wheelchair curling (2006–2014). Para snowboard made its Winter Paralympic Games debut as a discipline under para alpine skiing in 2014. The number of events contested in these sports in each edition of the Winter Paralympic Games is presented in Table 2. Overall, 739 of the 939 events contested between 1976 and 2014 (84%) have been in two sports, namely alpine skiing (49%) and cross country skiing (35%).

**Table 2:** Events contested by sport in the Winter Paralympic Games

Year	Alpine Skiing	Cross country skiing	Ice sledge speed skating	Biathlon	Ice sledge hockey	Wheelchair curling	Total
1976	28	25	–	–	–	–	53
1980	22	27	14	–	–	–	63
1984	56	35	16	–	–	–	107
1988	43	38	12	3	–	–	96
1992	48	27	–	4	–	–	79
1994	66	48	8	10	1	–	133
1998	54	39	16	12	1	–	122
2002	53	32	–	6	1	–	92
2006	24	20	–	12	1	1	58
2010	30	20	–	12	1	1	64
2014	32	20	–	18	1	1	72
Total	456	331	66	77	6	3	939

Even though home advantage in para sports has not been investigated thus far, it has been documented to a certain extent in specific winter sports among non-disabled athletes. Bray and Carron found some evidence of home advantage in elite-level alpine skiing including statistical significance on some measures [18]. A subsequent study by Balmer et al. examined home advantage in the Winter Olympic Games from 1908 to 1998 [13]. Their study also reported evidence of home advantage in alpine skiing, which reinforces the findings from Bray and Carron’s study. Figure skating, freestyle skiing, ski jumping and short track speed skating were the other sports found by Balmer et al. to exhibit a significant home advantage. On the other hand, they found little or no home advantage in cross country skiing, biathlon, ice hockey and speed skating amongst other sports (Nordic combined, bobsled and luge). When events were grouped according to whether they were subjectively assessed by judges, significantly greater home advantage was observed in the subjectively assessed events (figure skating and freestyle skiing) than other events (*p* < 0.05), suggesting that judges were scoring home competitors disproportionately higher than away competitors [13]. Home advantage in subjectively assessed events has also been shown to exist in other international multi-sport competitions featuring summer sports [6, 16, 17]. However, none of the sports in the Winter Paralympic Games programme between 1976 and 2014 were reliant on subjective scoring by judges.

Following the Balmer et al. study [13], attempts to examine home advantage in international competitions that feature winter sports have been few and far between. Koning analysed elite speed skating data from World Cups, World Championships and the Winter Olympic Games from 1986 to 2003 and found that a competitor skated faster at home than in another country, although the magnitude of the home advantage was very small [14]. To the best of our knowledge, there has been no formal investigation into home advantage in the sport of curling. Recently, Pettigrew and Reiche used a linear regression model to examine the size of the home advantage effect at country level in the Winter Olympic Games over 17 editions between 1952 and 2014 [12]. While this study showed that host countries tend to increase their number of gold medals by around two and their total medal count by around four compared to the Games prior to hosting, neither of the results were found to be statistically significant at conventional levels. Our research is the first attempt to directly measure the size of the home advantage in the Winter Paralympic Games. The objectives of the research were as follows:

- To analyse the overall performance of host nations in the Winter Paralympic Games when competing at home and away from home.
- To examine sport-specific variations in home advantage in the Winter Paralympic Games.
- To compare the size of the home advantage effect in the Winter Paralympic Games with the Winter Olympic Games.

## Methods

The results of each edition of the Winter Paralympic Games between 1976 and 2014 were sourced from the official website of the Paralympic Movement (https://www.paralympic.org/results/historical) and recorded in SPSS (version 24). As illustrated by the data presented in Table 2 previously, there has been considerable fluctuation in the total number of events contested in the Winter Paralympic Games over time, ranging from a high of 133 in 1994 to a low of 53 in 1976. The number of events contested within the sports of alpine skiing (22–66), cross country skiing (20–48), biathlon (3–18) and ice sledge speed skating (8–16) has also not been the same throughout. Therefore, using absolute measures of performance such as the gold medal count or the total medal count does not control for the number of medals on offer or for the performance of non-hosting nations. For these reasons, we measured performance by: first, converting the number and type of medals won by each nation in a given edition into points (gold = 3, silver = 2 and bronze = 1); and second, expressing those points as a proportion of the total number of points won by all competing nations in that edition. This performance measure is termed market share. For example, in 2006 the host nation—Italy—won 14 medal points out of 348 medal points awarded and their overall home edition market share was, therefore, 4.02% (i.e. 14 divided by 348).

Table 3 shows the number of valid home and away observations for each host country. To obtain a measure of home advantage, we first compared each nation’s average home performance with its own average away performance. For example, Italy’s home average market share of 4.02% was compared with its own average away market share (across nine editions) of 1.85%. This approach ensured that less successful countries were not unfairly compared with more successful countries. Countries that did not host the Winter Paralympic Games were excluded from the analysis. This was because they had no home performances to compare with their away performances.

**Table 3:** Valid home and away observations for each host nation in the Winter Paralympic Games

Country	Home		Away	
	Number	Years	Number	Years
Sweden	1	1976	10	1980–2014
Norway	2	1980, 1994	9	1976, 1984–1992, 1998–2014
Austria	2	1984–1988	9	1976, 1992–2014
France	1	1992	10	1976–1988, 1994–2014
Japan	1	1998	10	1976–1994, 2002–2014
USA	1	2002	10	1976–1998, 2006–2014
Italy	1	2006	9	1980–2002, 2010–2014
Canada	1	2010	10	1976–2006, 2014
Russia	1	2014	5	1994–2010

For each host nation, we also compared its home market share with its average market share in the editions immediately before hosting and immediately after hosting. For example, Italy’s market share in 2002 (pre-home) and 2010 (post-home) was 3.26 and 3.12%, respectively—an average of 3.19%. Therefore, its performance at home in 2006 was 0.83% points better than its average pre/post-home performance (i.e. 4.02 minus 3.19%). In instances where there was no valid pre-home or post-home data (i.e. pre-1976 for Sweden; post-1984 and pre-1988 for Austria; post-2014 for Russia), only the available away (pre or post) data point is utilised in the home advantage calculation. The number of countries included in the analysis varied for each sport, since not all sports have been contested in each edition of the Winter Paralympic Games. For example Sweden, the 1976 host, was eliminated from the biathlon analysis because biathlon was introduced in 1988 and hence there was no home data point to compare with away data points.

Selected comparisons at country level and sport-specific level are also drawn with the Winter Olympic Games. The results of the Winter Olympic Games were obtained from https://www.olympic.org/olympic-results and the time period chosen for this analysis was 1992–2014. The rationale for this restriction was that 1992 was the point from which both the Winter Olympic Games and the Winter Paralympic Games have been hosted by the same nations and in the same years. For this comparative analysis, the data for the sports of alpine skiing, cross country skiing and biathlon are based on six observations each (1992–2014) whereas the scores for ice hockey (1996–2014), curling (2006–2014) and speed skating (1994–1998) are based on five, three and two data points respectively, based on the availability of matched pairs.

In consideration of the limited number of observations available, a Wilcoxon signed rank test was used to determine whether there was a genuine difference in nations’ performance under host and non-host conditions. A Spearman’s rank-order correlation was run to assess the relationship between team quality and home advantage at country level.

## Results

### Performance of host nations in the Winter Paralympic Games

**Fig. 1:**
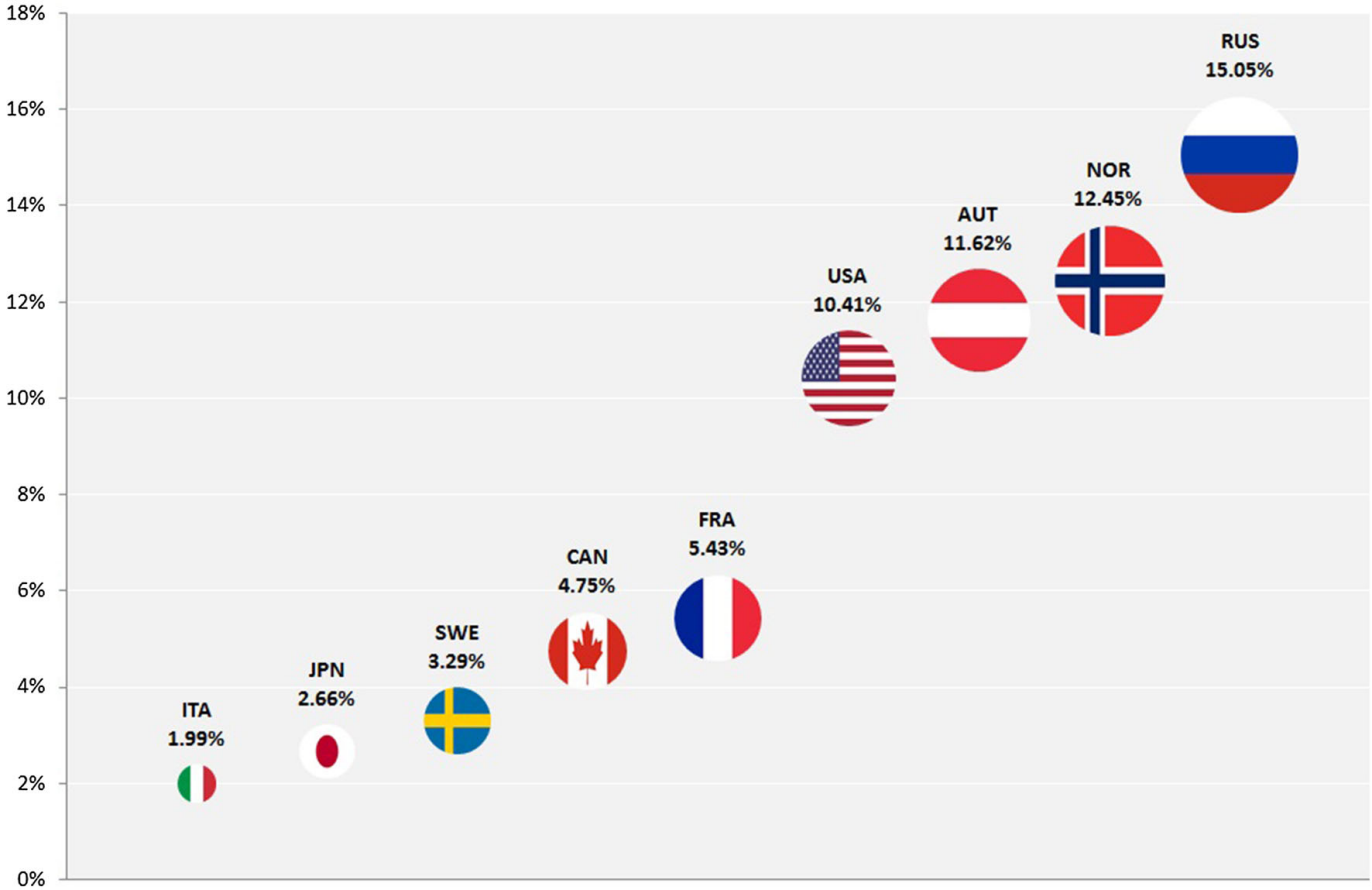
Overall market shares of host nations in the Winter Paralympic Games 1976–2014

**Fig. 2:**
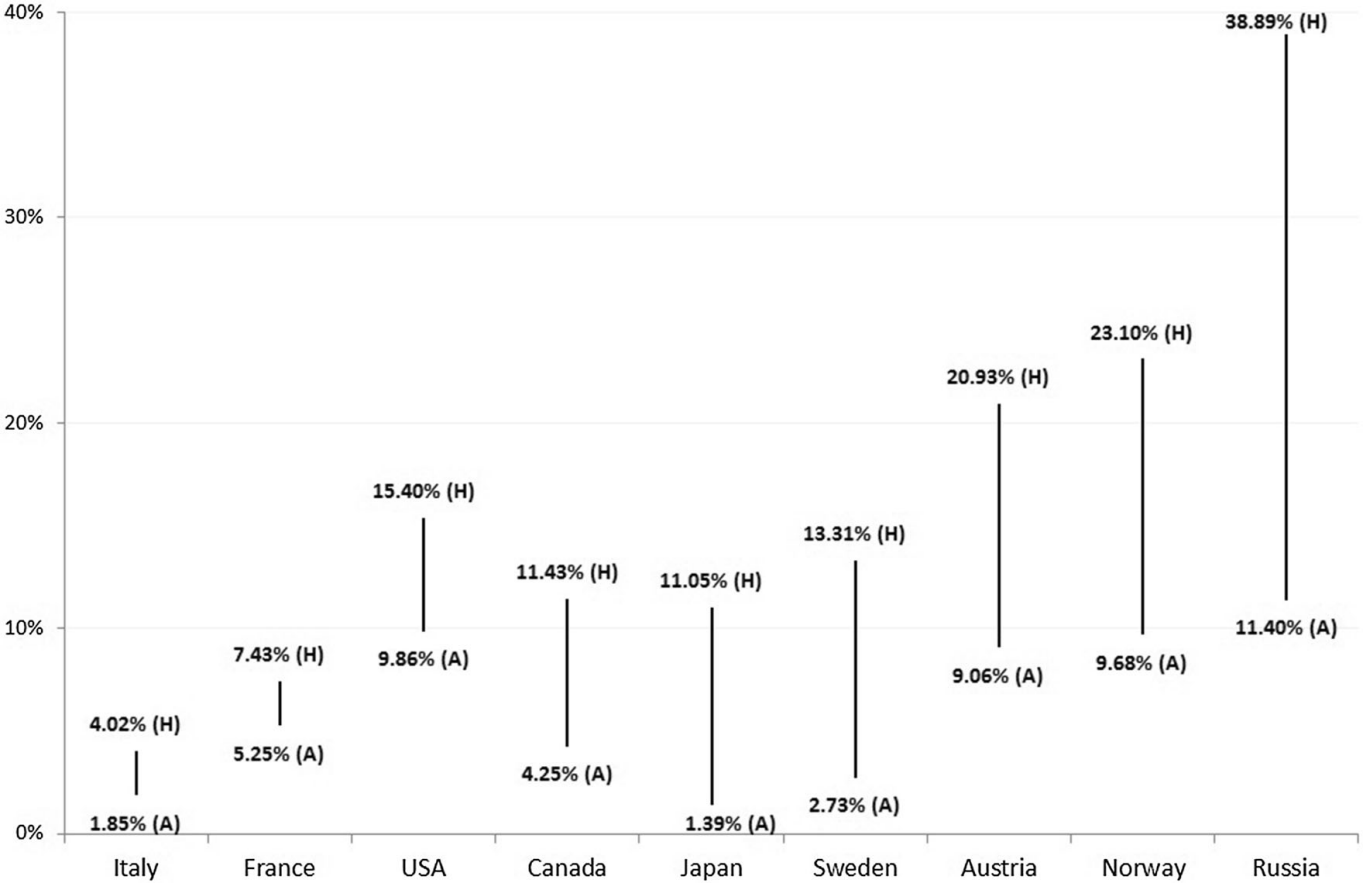
Average home (*H*) and away (*A*) market shares of host nations in the Winter Paralympic Games 1976–2014

The overall market share of the nine host nations in the Winter Paralympic Games from 1976 to 2014 is presented in Fig. 1. The overall level of success achieved in this time frame varies considerably by nation. The data in Fig. 1 do not differentiate between home and away performances. This differentiation is shown in Fig. 2, which compares the home market share performance with the away market share performance.

The key point from Fig. 2 is that each nation’s average home market share exceeds its average away market share. The magnitude of the difference ranges between 2.17% points in the case of Italy to 27.49% points for Russia. Using a Wilcoxon signed rank test on two sets of nine observations, the difference between nations’ home and away performance was found to be significantly greater than zero (*z* = −2.666, *p* = 0.008), which suggests that there is a genuine home advantage effect.

Because we are comparing performance typically in a single home edition (two in the case of Norway and Sweden) with pooled data for multiple away editions (between five and ten) over a long time period (nearly four decades), it is possible that the estimates of home advantage in Fig. 2 might be somewhat biased. To account for any fluctuations in performance of nations over time, Table 4 compares the home market share of each host nation in every edition to its own away market share in the editions immediately before (pre-home) and after (post-home) hosting the competition. There is no away comparator for Sweden pre-1976 and for Russia post-2014. Austria hosted two consecutive editions in 1984 and 1988—hence there are no valid post-home and pre-home (away) data points respectively in these instances.

**Table 4:** Pre-home, home and post-home market shares of host nations in the Winter Paralympic Games 1976–2014

Year	Host	Pre-home (%)	Home (H) (%)	Post-home (%)	Avg. pre/post-home (PPH) (%)	Difference (H − PPH) (%)
1976	Sweden	NA	13.31	8.33	8.33	4.98
1980	Norway	9.90	34.77	13.23	11.56	23.21
1984	Austria	12.64	24.72	NA	12.64	12.08
1988	Austria	NA	16.67	8.28	8.28	8.39
1992	France	4.96	7.43	8.14	6.55	0.88
1994	Norway	6.16	18.02	11.60	8.88	9.15
1998	Japan	1.13	11.05	0.54	0.83	10.22
2002	USA	9.28	15.40	8.05	8.66	6.74
2006	Italy	3.26	4.02	3.12	3.19	0.83
2010	Canada	7.47	11.43	7.41	7.44	3.99
2014	Russia	20.26	38.89	NA	20.26	18.63

When comparing the market share of host nations at home with their average performance in the immediate pre-home and post-home (away) editions, we found that all previous host nations performed better at home. The difference between the mean home market share (17.79%) and the mean away market share (8.78%) using this approach was 9.01% points, which is slightly less than the mean differential obtained by comparing nations’ home performances with all their away performances (10.01% points). However, our results still suggest that when nations compete on home soil in the Winter Paralympic Games, their performance in terms of market share improves. The Wilcoxon signed rank test confirms that the observed difference between home and away performances was significant (*z* = −2.934, *p* = 0.003). If we accept away performance to be a reliable indicator of team quality, then there is a strong positive correlation between host nations’ average away market share and the size of the home advantage effect (*r*_s_ = 0.691, *p* = 0.019).

### Sport-specific findings

Table 5 shows the differences between nations’ performance at home and their average pre/post-home performance for each Winter Paralympic sport. Only alpine skiing (*z* = −2.395, *p* = 0.017) and cross country skiing (*z* = −2.401, *p* = 0.016) returned statistically significant diff

**Table 5:** Difference between home and average pre/post home performances of host nations in the Winter Paralympic Games by sport

Year	Host	Alpine skiing (%)	Cross country skiing (%)	Ice sledge speed skating (%)	Biathlon (%)	Ice sledge hockey (%)	Wheelchair curling (%)
1976	Sweden	0.00	9.85	NA	NA	NA	NA
1980	Norway	5.00	14.29	23.00	NA	NA	NA
1984	Austria	−2.07	20.26	2.15	NA	NA	NA
1988	Austria	12.09	2.82	NA	11.11	NA	NA
1992	France	−0.38	1.70	NA	−6.67	NA	NA
1994	Norway	3.35	7.00	62.50	12.22	−16.67	NA
1998	Japan	1.95	1.02	64.58	6.94	0.00	NA
2002	USA	5.51	1.87	NA	0.00	41.67	NA
2006	Italy	6.34	−4.06	NA	−1.39	0.00	0.00
2010	Canada	9.49	0.83	NA	−2.07	−33.33	0.00
2014	Russia	17.80	16.67	NA	13.80	33.33	33.33

erences between home and away performances.

### Comparison with the Winter Olympic Games

Table 6 compares the magnitude of the home advantage effect in the Winter Paralympic Games with the Winter Olympic Games for the seven nations that have hosted the competitions between 1992 and 2014. The average home advantage effect in this time frame for the Winter Paralympic Games is 7.20% points compared with 3.71% points in the case of the Winter Olympic Games. The difference between the scores for the Winter Paralympic Games and the Winter Olympic Games is not significant (*z* = −1.690, *p* = 0.091).

**Table 6:** Home advantage in the Winter Paralympics versus the Winter Olympics 1992–2014

Host	Winter Paralympics			Winter Olympics		
	Home (%)	Avg. pre/post-home (%)	Difference (%)	Home (%)	Avg. pre/post-home (%)	Difference (%)
France	7.43	6.55	0.88	5.83	1.54	4.29
Norway	18.02	8.88	9.15	15.57	13.10	2.47
Japan	11.05	0.83	10.22	5.11	1.55	3.56
USA	15.40	8.66	6.74	14.26	8.57	5.69
Italy	4.02	3.19	0.83	4.17	3.43	0.73
Canada	11.43	7.44	3.99	11.80	9.42	2.38
Russia	38.89	20.26	18.63	11.86	5.03	6.84

**Fig. 3:**
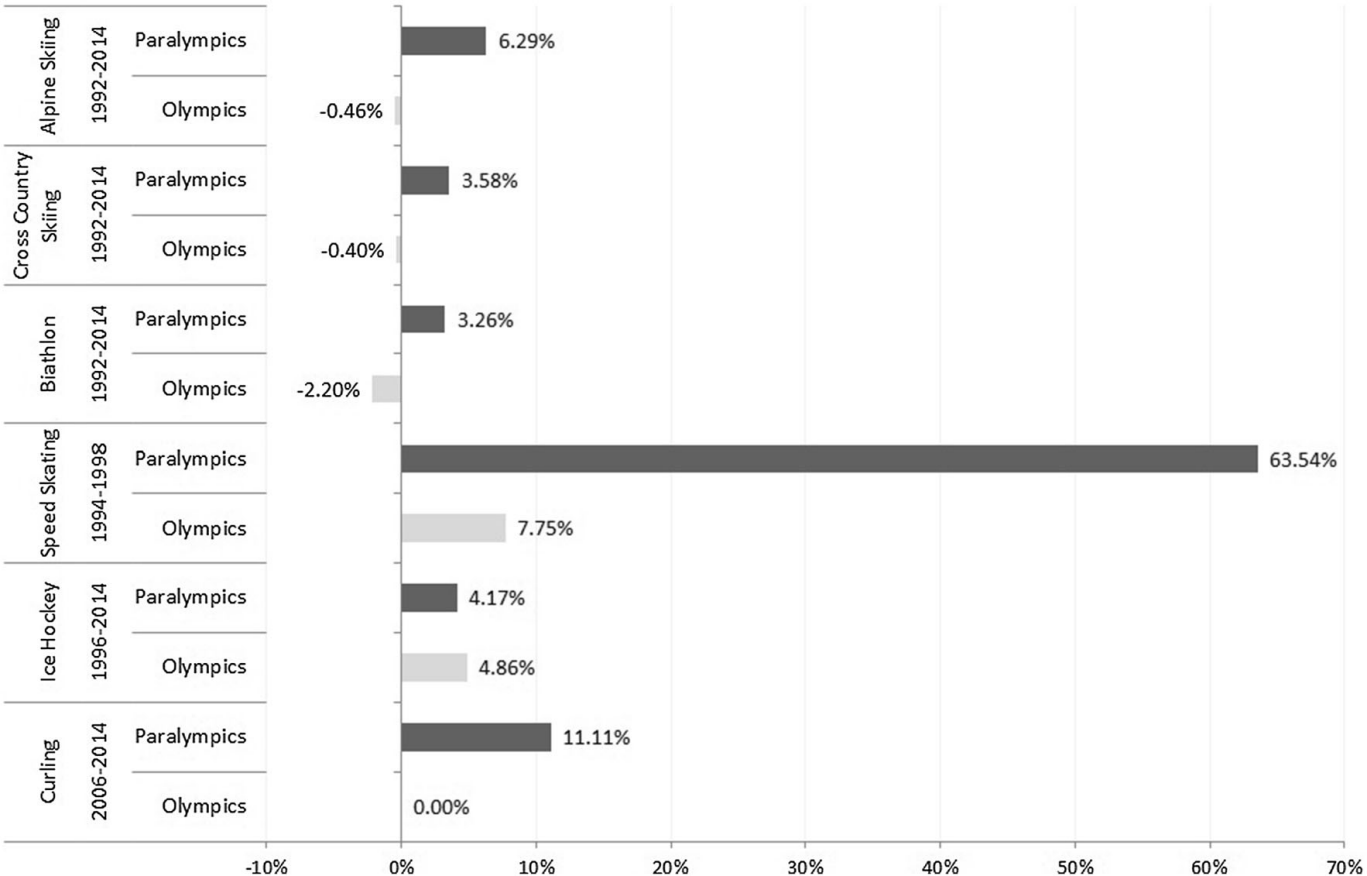
Mean difference between home and average pre/post-home performance by sport in the Winter Paralympics and the Winter Olympics

Figure 3 shows the direction and magnitude of the home advantage effect in the Winter Olympic Games and Winter Paralympic Games by sport. With the exception of ice hockey, the difference between home performance and average pre/post-home performance is greater in the case of the Winter Paralympic Games. However, given the small sample sizes involved none of these differences were found to be statistically significant (*p* > 0.05).

## Discussion

The academic literature on home advantage in sport can be categorised along two broad lines: (1) descriptive research, which focuses on investigating the prevalence and magnitude of home advantage in different sporting contexts; (2) explanatory research that examines the factors that contribute to home advantage. While home advantage is known to exist in professional team sports [4] and, to a lesser extent in individual sports [5], the extent to which home advantage exists in para sports is not known. With this in mind, our study was concerned primarily with the determination of home advantage in para sports. With reference to the theoretical model for home advantage research advanced by Courneya and Carron [1] and its refinement by Carron et al. [2] in Table 1, we considered the impact of game location on performance outcomes in the Winter Paralympic Games between 1976 and 2014.

Our analysis shows that host nations in the Winter Paralympic Games performed considerably better at home than away from home and that the difference between home and away performances was statistically significant (*p* < 0.05). In other words, strong evidence of a home advantage effect was identified. This finding resonates with previous research in the context of the Winter Olympic Games between 1908 and 1998 [13]. Pettigrew and Reiche [12] also examined home advantage in the Winter Olympic Games between 1952 and 2014, although they reported that the home advantage effect at country level in this time frame was not statistically significant. We found that the size of the home advantage effect is significantly correlated with the quality of the host nation. This finding indicates that home advantage is typically larger in the case of stronger nations.

Our analysis also points to sport-specific variations in home advantage in the Winter Paralympic Games. Across the six sports to be held in the Winter Paralympic Games to date, only alpine skiing and cross country skiing exhibited a significant home advantage effect (*p* < 0.05). The prevalence of home advantage in alpine skiing in our study is in line with previous evidence from the Winter Olympic Games [13] and World Cups [18]. In contrast, while cross country skiing exhibited a significant home advantage effect in our study, a previous study did not find any evidence of home advantage in this sport among able-bodied athletes [13]. Building on recent research [19, 20] future studies should attempt to better understand the relative importance of game location within particular competition phases of these sports to provide more technical and tactical references for coaches, who regularly try to benefit from valuable information in planning training and competition. Evidence of home advantage in the remaining Winter Paralympic sports of biathlon, curling, ice sledge hockey and ice sledge speed skating was either weak or inconclusive.

Home advantage in some international multi-sport events has been documented in sports that require subjective judgments [13, 16, 17]. However, neither cross country skiing nor alpine skiing are reliant on subjective scoring by judges and none of the other four para sports that have featured at the Winter Paralympic Games were found to have a significant home advantage. Therefore, future research should investigate the game location factors that influence home advantage in the competition and how they affect the psychological, physiological and behavioural states of competitors and coaches. Based on previous research, potential factors that may elevate the performance of competitors when competing at home include: learning factors (i.e. familiarity with the venue), particularly in alpine skiing [13, 18]; higher testosterone levels [21]; the absence of jet lag associated with travel [22].

When comparing home advantage in the Winter Paralympic Games with the Winter Olympic Games for the last seven host nations (1992–2014), we found that host nations typically performed better at home in both competitions in this time frame and that home advantage was generally more pronounced (albeit not a statistically significant difference) in the case of the Winter Paralympic Games. The reasons underpinning the differences in the magnitude of home advantage between the two competitions at both country level and sport-specific level are worthy of further investigation.

## Conclusion

This research has extended the evidence base of home advantage in international multi-sport events and, to the best of our knowledge, is the first formal attempt to examine home advantage in the context of a para sport competition. In summary, there is clear evidence of a home advantage effect in the Winter Paralympic Games at country level and its magnitude appears to be greater than in the Winter Olympic Games. In addition to investigating the factors that contribute to these findings, future research should apply similar methods to the Summer Paralympic Games, which incorporates more sports and events.
